# Supplementary material for: Structural basis for the clamping and Ca2+ activation of SNARE-mediated fusion by synaptotagmin
Source: Nat Commun. 2019 Jun 3;10:2413. doi: 10.1038/s41467-019-10391-x (PMC6546687; doi:10.1038/s41467-019-10391-x)
Supplement: Supplementary file 3 — Description of Additional Supplementary Files [file 41467_2019_10391_MOESM3_ESM.pdf]

Description of Additional Supplementary Files

File Name: Supplementary Movie 1

Description: Fitting of the separate C2A (magenta), C2B (grey) and SNARE (multi-color) derived from the Syt1-SNARE crystal structure (PDB code: 5CCI) onto the cryo-EM map shows that the Syt1 C2B domain concurrently binds both the SNARE proteins and the lipid membranes (yellow) on diametrically opposite surfaces under resting (1mM Mg2+-conditions).
