# Supplementary material for: Structural basis for the clamping and Ca2+ activation of SNARE-mediated fusion by synaptotagmin
Source: Nat Commun. 2019 Jun 3;10:2413. doi: 10.1038/s41467-019-10391-x (PMC6546687; doi:10.1038/s41467-019-10391-x)
Supplement: Supplementary file 2 — Peer Review File [file 41467_2019_10391_MOESM2_ESM.pdf]

Editorial Note: This manuscript has been previously reviewed at another journal that is not operating a transparent peer review scheme. This document only contains reviewer comments and rebuttal letters for versions considered at Nature Communications.

REVIEWERS' COMMENTS:

Reviewer #1 (Remarks to the Author):

I would like to thank the authors for addressing my concerns. However, I would like to ask that proper reference will be given to reference 48 (Brunger et al., Trends in Cell Biology) and reference 42 (Zhou et al., Nature, 2017) in the caption of Figure 6. The overall idea in figure 6 is very similar to the model shown in reference 48, Figure 3 "Core arrangement".

Reviewer #2 (Remarks to the Author):

This manuscript has been carefully revised in light of the suggestions made during the first round of review. It is now much clearer and more convincing than before; in addition, I think it is also more balanced. The results are interesting and move the field forward. I recommend publication.
